# Investigation of annealing effects on the physical properties of Ni_0.6_Zn_0.4_Fe_1.5_Al_0.5_O_4_ ferrite

**DOI:** 10.1039/c9ra02238d

**Published:** 2019-06-26

**Authors:** R. Lahouli, J. Massoudi, M. Smari, H. Rahmouni, K. Khirouni, E. Dhahri, L. Bessais

**Affiliations:** Unité de recherche Matériaux Avancés et Nanotechnologies (URMAN), Institut Supérieur des Sciences Appliquées et de Technologie de Kasserine, Université de Kairouan BP 471 Kasserine 1200 Tunisia rahmounihedi@yahoo.fr rahmounihedi@gmail.com; Laboratoire de Physique Appliquée, Faculté des Sciences, Université de Sfax B. P. 1171 Sfax 3000 Tunisia; Laboratoire de Physique des Matériaux et des Nanomatériaux appliquée à l'Environnement, Faculté des Sciences de Gabès cité Erriadh, Université de Gabès 6079 Gabès Tunisia; ICMPE (UMR 7182), CNRS, UPEC, Université Paris Est 94320 Thiais France

## Abstract

In the present study, the structural, morphological, electrical, and dielectric properties of Ni_0.6_Zn_0.4_Fe_1.5_Al_0.5_O_4_ annealed at 600 °C, 900 °C, and 1200 °C were investigated. The X-ray diffraction patterns confirmed the presence of the single-phase cubic spinel structure with the *Fd*3̄*m* space group. The SEM images of Ni_0.6_Zn_0.4_Fe_1.5_Al_0.5_O_4_ nanoparticles demonstrated that these samples (Ni900 and Ni1200) were nano-sized and that the increase in annealing temperature enhanced the agglomeration rate. It was found that the electrical conductivity of the system improved on increasing the temperature over the whole explored range for the two low annealing temperatures, while this improvement declined after 500 K in the case of the highest annealing temperature. For such a sample, a metallic behavior was seen. The sample annealed at 1200 °C possessed the highest conductivity and the lowest activation energy. The impedance measurements were in good agreement with the conductivity plots and confirmed the emergence of a grain boundary effect with the increase in annealing temperature. For the sample annealed at the highest temperature, *Z*′ decreased rapidly with frequency. This sample exhibited the lowest defect density than the other samples. Consequently, its electrical conductivity increased. A Nyquist diagram was used to examine the contribution of the grains and grain boundary to conduction and to model each sample by an equivalent electrical circuit. The dielectric behavior of the investigated samples was correlated to the polarization effect.

## Abstract 2

In the present study, the structural, morphological, electrical, and dielectric properties of Ni_0.6_Zn_0.4_Fe_1.5_Al_0.5_O_4_ annealed at 600 °C, 900 °C, and 1200 °C were investigated.

## Graphical abstract

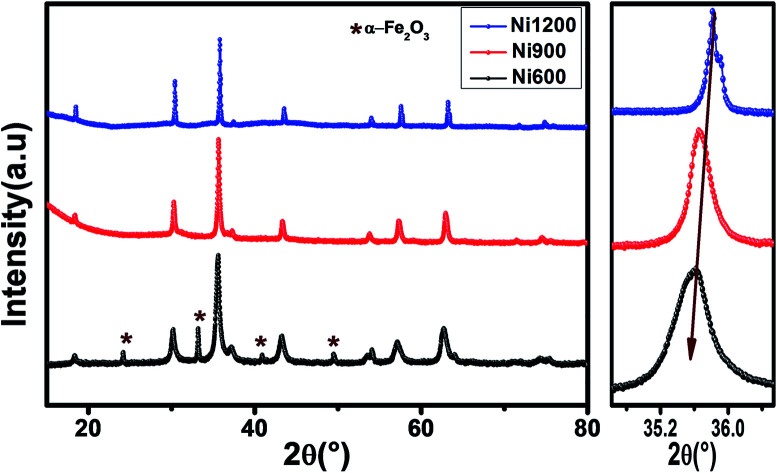

## 1. Introduction

The Scientific Committee has been concerned extensively regarding ferrites, which have been exploited for the past five decades.^1^ They constitute a family of magnetic materials that are of great interest because of their importance in the fundamental understanding of physical processes and their technological applications in various fields.^2^ Indeed, to be classified so, they take advantage of the fact that they can combine excellent electrical, magnetic, and chemical properties^3^ such as significant magnetic saturation, high electrical resistivity, low electrical losses, and very good chemical stability. They exist under three main types of structures: hexaferrites or hexagonal ferrites, rare earth-based ferrates or ferrites and spinel ferrites.^4^ This study focuses on the last family.

Spinel ferrites have utility in several fields. Hence, they are used as key materials in the enhancement of the information storage area, magnetic refrigeration, the technology of ferrofluids,^5,6^ and as gas sensors.^7,8^ Recently, they have been used as photocatalysts in the detoxification of biological fluids.^5,6^ Thus, spinel ferrites are used to purify water and disinfect the atmosphere^9–11^ by bombarding the organic-compacted tangled mass that contaminates the environment and mineralizing them by degradation to water and CO_2_.^12^ Understanding their physical characteristics and special structural and electrical properties is a crucial step to profit in the best way from their scientific values. For this reason, many researchers have explored these materials. It has been found that they are oxide semi-conductors of the general formula MFe_2_O_4_ (M: transition metals: Ni, Co, Mg, Zn, *etc.*). The occurrence of oxygen is one of the most important properties that makes them suitable for photocatalysis, a promising field of application. Photocatalysis, a technology of advanced oxidation, requires light, which excites an electron from the valence band to the conduction band, leaving behind a hole in the valence band. The electron–hole pairs allow the capture of free radicals, which react with the compounds present in the environment and promote the process of oxidation and reduction.^12^ Thus, we should rather slow down their recombination. It is a phenomenon that involves the external layer, in which absorption/desorption occurs. In addition to the presence of oxygen, spinel ferrites have large band gaps, which limit their activity to a UV light. In order to widen this range of activity and to improve their photocatalytic performances, some doping elements can be included to minimize the band gap. Then, the domain of activity will be extended to all the ranges of visible light.^13,14^

Considering the usefulness of spinel ferrites, numerous methods of fabrication were used to obtain these materials tailored to expectations. These methods include forced hydrolysis in a polyol,^15^ reverse micelle,^16^ shock wave,^17^ mechanical alloying,^18^ synthesis from a citrate precursor,^19^ sonochemical,^20^ hydrothermal,^21^ co-precipitation,^22^ and sol–gel.^23^ Obviously, the most useful and attractive technique is the sol–gel method due to its various advantages such as the production of ultrafine particles with narrow size distribution without the requirements of a long processing time and considerable thermal energy, good stoichiometric control,^23–25^ and high crystalline quality of the products obtained by this method.^25^ From the literature related to synthesis methods, ionic substitutions are the major factors that influence the physical and chemical properties of nanoferrites.^26^ Besides, the sintering temperature is also an important parameter;^26^ its effect has been widely explored but its impact is not standard and depends on the ferrite composition. In general, the annealing conditions influence the magnitude of conductivity and the dielectric behavior of ferrites.^27^

Ni-ferrites have an inverse spinel structure, while Zn-ferrites crystallize in a normal spinel structure. Combining these two structures by studying Ni–Zn ferrites offers an opportunity to benefit from their special properties;^28^ they show promising applications in radio frequency circuits, non-resonant devices, high-quality filters, transformer cores, rod antennas, and read-write heads for high-speed digital tapes and operating devices.^29–31^ In this paper, the effect of the annealing temperature on the structural, morphological, electrical, and dielectric properties of Ni_0.6_Zn_0.4_Fe_1.5_Al_0.5_O_4_ was evaluated. To the best of our knowledge, this is the first reported study on the effect of the above-mentioned factors on the behavior of this latter compound. Thus, it is an occasion to advance a close treatment of this subject and evaluate the suitability of this material to different applications.

## 2. Experimental

Ni_0.6_Zn_0.4_Fe_1.5_Al_0.5_O_4_ nanoparticles were prepared by the sol–gel method. Analytical purity grade Fe(NO_3_)_3_·9H_2_O and NiCl_2_·6H_2_O (Sigma-Aldrich) were dissolved in distilled water. The oxide powders ZnO and Al_2_O_3_ with high purity (99.99%) were dissolved in nitric acid and added drop-wise to a solution and mixed with citric acid (C_6_H_8_O_7_) (all delivered by Sigma Aldrich) with a molar ratio of 1 : 1.5 of metal cation (Ni + Fe + Zn + Al) to citric acid, followed by the addition of ethylene glycol (C_2_H_6_O_2_). During this procedure, the resulting solutions were continuously stirred using a magnetic agitator at 80 °C to remove excess water and to form a gel. Then, they were heated to 300 °C while annealing constantly to transform into a xerogel. Finally, the samples were pelletized and annealed at 500 °C for 6 h in an electrical muffle furnace and cooled slowly to room temperature. The product was pelletized at a pressure of 6 tons per cm^2^ and annealed at 600 °C (Ni600) and 900 °C (Ni900) for 6 h and at 1200 °C (Ni1200) for 24 h to obtain samples with different particle sizes.

The homogeneity and phases purity of the samples were characterized by X-ray diffraction (XRD) at room temperature using a BRUKER diffractometer with CuKα radiation (*λ* = 1, 5406 Å). The data acquisition was in the 2*θ* range of 15–80°, with a step of 0.02° and an acquisition time for each step of 1 s. The XRD data were used to obtain the lattice structure and cell parameters of the synthesized compounds by means of Rietveld analysis using the FULLPROF program.

Microstructures and grain sizes were observed by a Merlin scanning electron microscope (SEM) equipped with an Oxford Instruments silicon drift detector (SDD)-X-Max 50 used for the elemental analysis of various phases.

The electrical characterization was assured by impedance spectroscopy using an Agilent 4294A impedance analyzer. Before starting this characterization, the samples must have the form of dipoles; thus, we deposited a silver thin film of some nanometers on each pellet's face using a vacuum thermal evaporator. Then, we stuck a silver wire using silver lacquer on each side. The explored range of frequency was between 40 Hz and 110 MHz. The temperature was from 300 K to 600 K. The samples were characterized under an excitation alternative signal of 50 mV.

## 3. Results and discussion

### 3.1 Structural properties

**Fig. 1:**
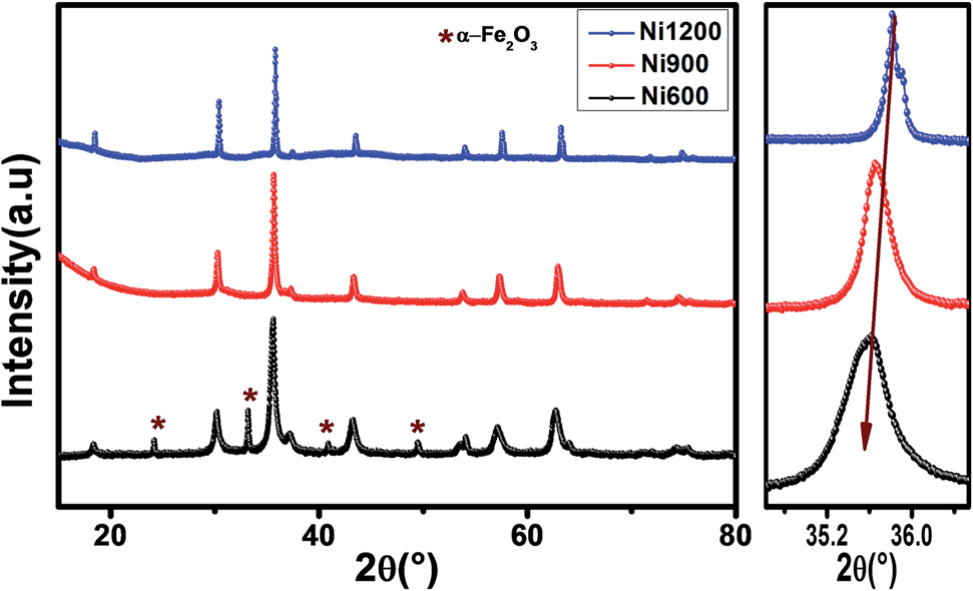
XRD patterns of Ni_0.6_Zn_0.4_Fe_1.5_Al_0.5_O_4_ annealed at different temperatures.

**Fig. 2:**
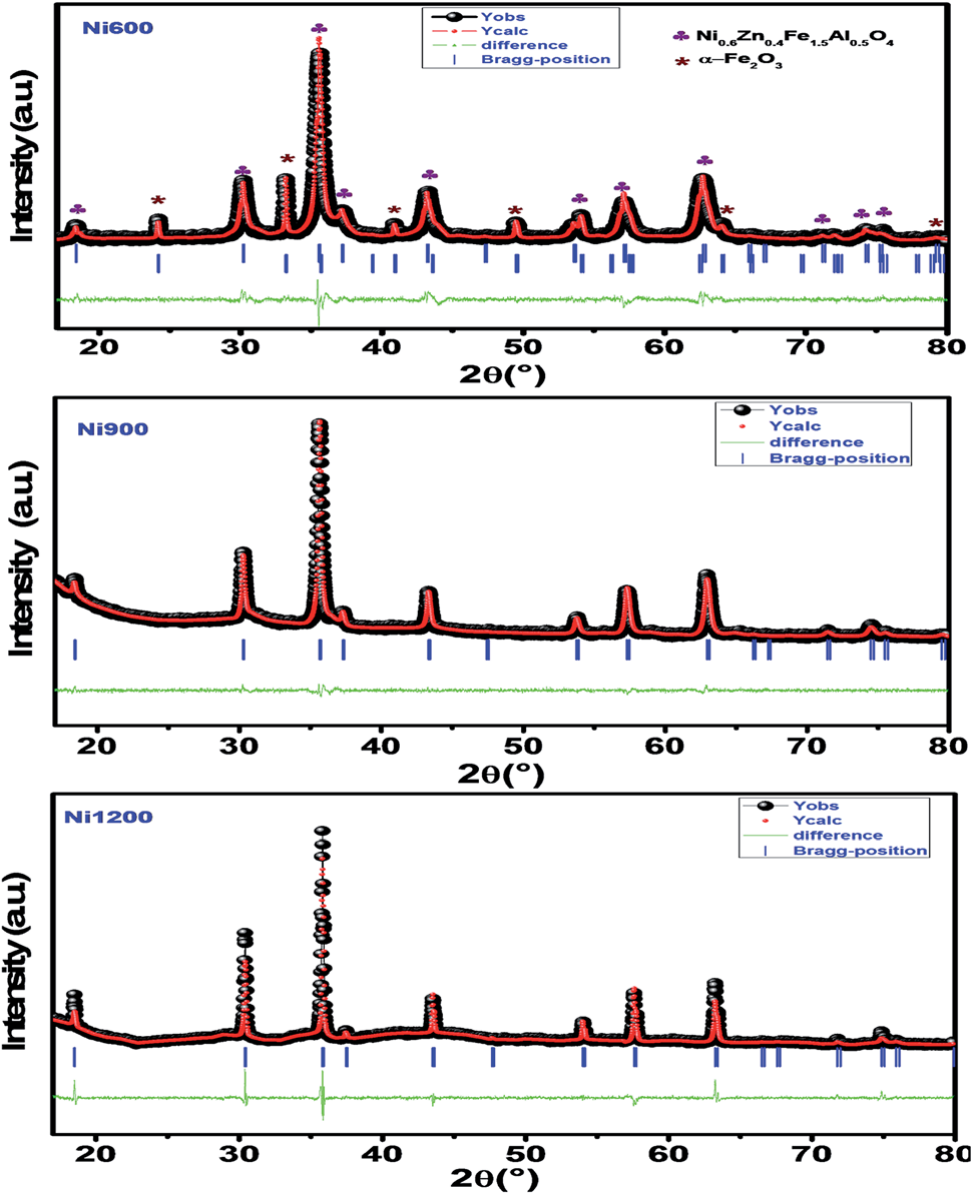
Rietveld-refined XRD patterns of Ni_0.6_Zn_0.4_Fe_1.5_Al_0.5_O_4_ annealed at different temperatures.

The X-ray diffraction patterns (Fig. 1) of the Ni_0.6_Zn_0.4_Fe_1.5_Al_0.5_O_4_ samples annealed at 600 °C, 900 °C, and 1200 °C exhibited the (111), (220), (311), (222), (400), (422), (511), (440), (620), (533), and (622) reflection planes, confirming the presence of the single-phase cubic spinel structure with the *Fd*3̄*m* space group. Additional peaks of the secondary phase (Fe_2_O_3_), designated by (*), were detected for the sample annealed at 600 °C (Ni600). The second phase of hematite was observed in the other ferrite compounds up to the annealing temperature of 900 °C.^32^ No diffraction peaks of other structures were detected in other samples, which indicated the homogeneity of the prepared samples. The high-temperature treatment remedies this second phase, and this was proven by the results in literature,^27,33,34^ which ensured that 1200 °C is sufficient to obtain a homogenous structure without any imperfections. The peak broadening observed in the XRD patterns of the samples annealed at 600 °C and 900 °C revealed the small crystallite size. According to the Scherrer's equation, 
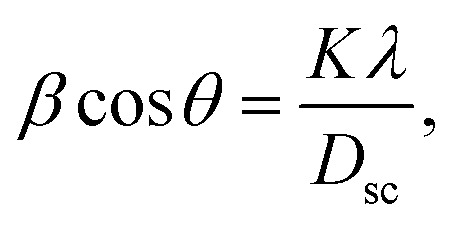
^13^ the most intense (311) reflection is used to determine the crystallite size of the samples. This was found to be at 17, 30, and 52 nm for the Ni600, Ni900, and Ni1200 samples, respectively. We can conclude that increasing the annealing temperature improves the crystallite growth. The highest value of 52 nm could be due to the increase in both the temperature and duration of annealing. The Rietveld-refined XRD patterns of the Ni600, Ni900, and Ni1200 samples are presented in Fig. 2. It is observed that all the experimental peaks are allowed Bragg 2*θ* positions (marked as vertical lines) for the *Fd*3̄*m* space group. In the refinement, the refined parameters were lattice parameters, scale factors, shape parameters, isotropic thermal parameters, and oxygen positions (*x* = *y* = *z*). The background was corrected by the pseudo-Voigt function. Various *R* factors are listed in Table 1. The values of *R*_p_ were found to be large. However, we observed a low value for the goodness of fit (*χ*^2^), which suggested that the refining of the samples was effective and that the samples obtained were of better quality. The lattice constant *a*_exp_ of the Ni–Zn nanoparticles was determined from X-ray data analysis using the relation 
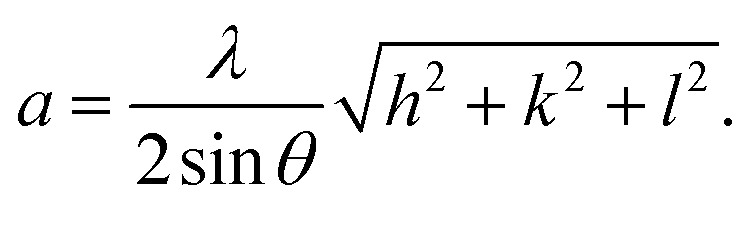
^24^ The crystallite size, lattice constant *a*_exp_, lattice constant *a*_r_ (obtained from Rietveld refinement), density, and porosity of the annealed samples are summarized in Table 1. It is evident from the table that the lattice constant continuously decreases with the increase in the sintering temperature. This decrease in the lattice constant may be due to the decrease in the particle surface stress and the higher crystal structure of larger particles. In addition, the decrease in lattice constant could be attributed to a finite size effect, cation/anion vacancies, and lattice stress due to reduction in the particle size.

**Table 1:** The lattice parameters, density, porosity, and crystallite size for Ni_0.6_Zn_0.4_Fe_1.5_Al_0.5_O_4_ at different annealing temperatures

	Ni_0.6_Zn_0.4_Fe_1.5_Al_0.5_O_4_ annealed at 600 °C	Ni_0.6_Zn_0.4_Fe_1.5_Al_0.5_O_4_ annealed at 900 °C	Ni_0.6_Zn_0.4_Fe_1.5_Al_0.5_O_4_ annealed at 1200 °C
*a*_exp_ (Å)	8.36851	8.32578	8.27865
*a*_r_ (Å)	8.37268	8.34727	8.31165
*R*_p_ (%)	20.9	24.9	51.8
*R*_wp_ (%)	13.1	11.1	21.8
*R*_e_ (%)	8.26	9.71	12.6
*χ* ^2^	2.490	1.297	3.001
*P* (%)	35.90424	34.93894	34.60559
*ρ*_X-ray_ (g cm^−3^)	5.04632	5.12441	5.21243
*D*_sc_ (nm)	17	30	52

**Fig. 3:**
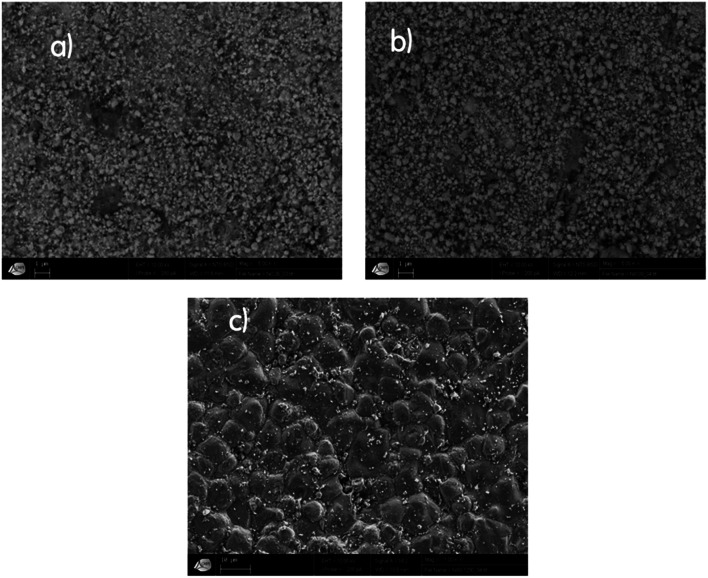
SEM images for Ni_0.6_Zn_0.4_Fe_1.5_Al_0.5_O_4_ annealed at 600 °C (a), 900 °C (b), and 1200 °C (c).

The morphology of our samples was observed with a scanning electron microscope (SEM), as illustrated in Fig. 3. The SEM micrograph of Ni1200 shows a polycrystalline structure. The grains have a polygonal shape with an average grain size *D*_SEM_ estimated at 4.6 μm, which is larger than the average crystallite size; this indicates that each grain observed by SEM is formed by several crystallites. The SEM images of Ni_0.6_Zn_0.4_Fe_1.5_Al_0.5_O_4_ nanoparticles demonstrate that these samples (Ni900 and Ni1200) are agglomerated and nano-sized. This observation was in good agreement with the increase in the crystallite size observed by XRD analysis and confirmed the good connectivity of grains. This crystallite growth will undoubtedly affect the conductivity and dielectric properties of the bulk sample.

### 3.2 DC conductivity and activation energy

**Fig. 4:**
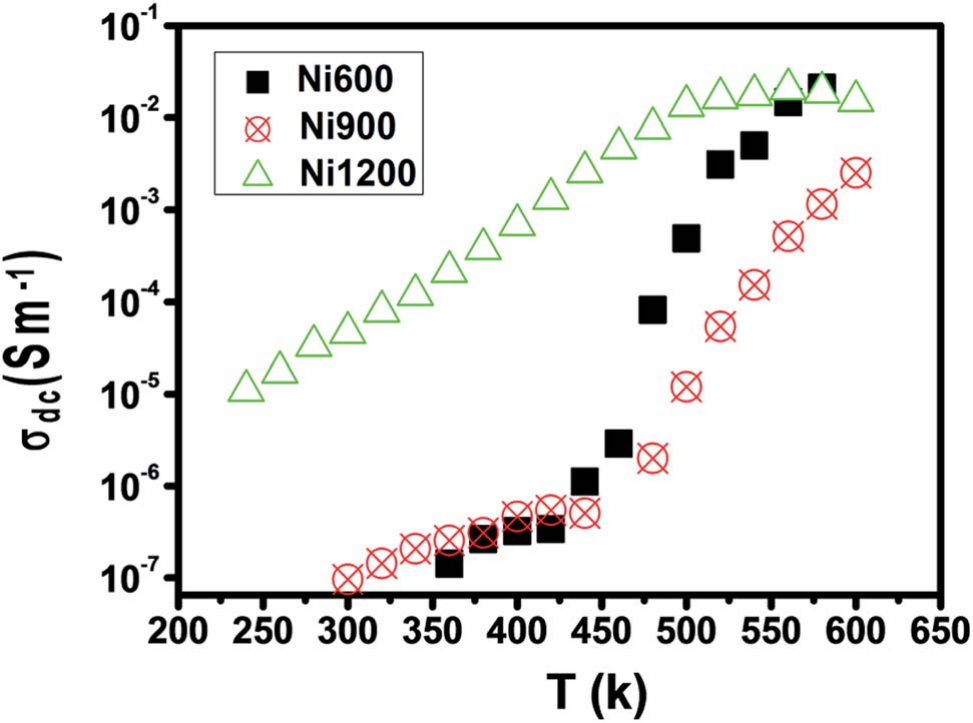
Plot of DC conductivity *versus* temperature for Ni_0.6_Zn_0.4_Fe_1.5_Al_0.5_O_4_ annealed at different temperatures.

The representation of the variation in conductivity with temperature on a direct current regime in Fig. 4 shows that the conductivity increases continuously with temperature for the samples annealed at 600 °C and 900 °C, which reflects a semiconductor behavior^35^ along the entire explored temperature range. In contrast, for the sample annealed at 1200 °C, the conductivity increased with temperature until 500 K; then, it became independent of the temperature, which indicated that saturation was reached. Knowing that the DC conductivity is a result of the magnitude of the density of free charge carriers and that charge carriers can be trapped by the defects in the material, such a behavior can be explained.^36^ As the temperature increases, traps emit charge carriers and the conductivity increases. For the sample sintered at 1200 °C, such emission stopped at 500 K when all the traps became empty and conductivity saturation was reached. We can conclude from this behavior that the sample annealed at 1200 °C contains lower defect density. It is also clear from this curve that the sample annealed at a higher temperature has higher DC conductivity. This can be due to the increase in the compactness of the specimens assured by increasing the annealing temperature.^37^ In general, the increase in conductivity with temperature is due to the fact that the drift mobility of charge carriers is thermally activated.

**Fig. 5:**
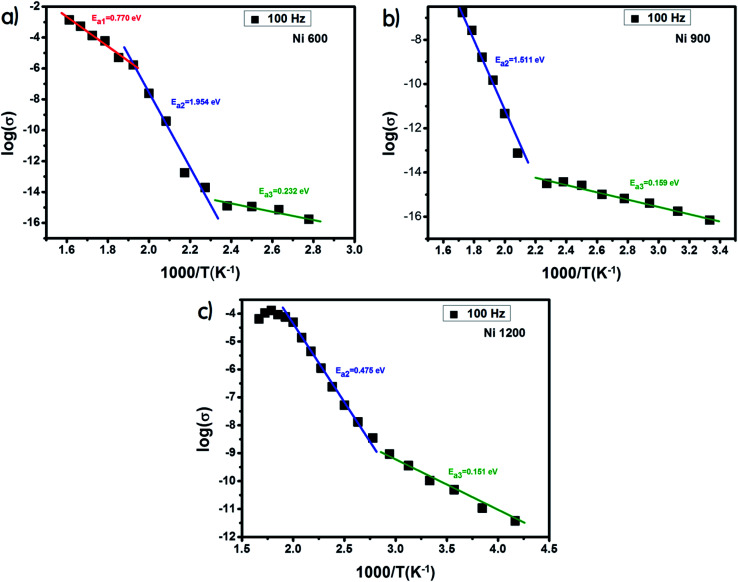
Plot of log(*σ*) *versus* temperature (10^3^/*T*) for Ni_0.6_Zn_0.4_Fe_1.5_Al_0.5_O_4_ annealed at 600 °C (a), 900 °C (b) and 1200 °C (c).

The examination of the variation in activation energies (summarized in Table 2, extracted from the Arrhenius plot shown in Fig. 5) with temperature allows noticing that the activation energy decreases with the increase in annealing temperature; thus its lowest value was attributed to the highest annealing temperature. This behavior is also found in the literature^38^ and it can be explained by the microstructural changes caused by the annealing conditions.^37^ Increasing the annealing temperature facilitates atom organization and reduces defect density, which can minimize the potential barrier that free carriers have to cross to participate in conductivity.^39^ According to E. M. M. Ibrahim,^39^ during the annealing process, some Zn ions undergo volatilization. Thus, in order to maintain global electrical neutrality, some Fe^3+^ ions are reduced to Fe^2+^ ions. These latter ions tend strongly to occupy B-sites.^40^ The hopping of electrons between Fe^3+^ and Fe^2+^ assures conduction and the increase in the annealing temperature increases the amount of Fe^2+^ ions in B-sites; thus, we suggest that the annealing process increases the hopping rate. Thereby, it minimizes the height of the electrical barrier crossed by charge carriers as the distance between two B-sites is the lowest compared to that between two A-sites or between an A-site and a B-site.^41^ In addition, according to the structural study, the crystallite size increases with the increase in the annealing temperature. Thus, the contact area between grains, which represents the electron path, increases, which implies a lower height of the potential barrier.^40,42–44^ Minimizing the potential barrier height implies minimizing the activation energy. For perovskite systems, the electrical properties intensely depend on the electron transfers inside the intragrain and intergrain. The electron transport in nonoperovskites is governed by the disorder defects created in grain boundaries region. To enhance the electrical conductivity in the grain boundary, it is necessary to research the modifying process of the grain boundary electrical transport with diverse average grain sizes.^45^ The latter influences the bulk, intergrain, total resistivity, and physical properties of the compound. In general, the electrical conductivity of the perovskite systems improves with annealing. The recrystallisation of grains during the annealing process and the tunneling of the charge carriers through the barriers of grain boundaries strongly affect the electrical transport mechanisms. The grain boundary model may be used to explicate the electrical conduction and charge transport in polycrystalline materials. For such a model, carrier transport will be reduced due to the encountered potential barriers and carrier mobility will be limited. Consequently, the grain size is considered as a significant factor that controls resistance. Recently,^46^ it was concluded that the crystallite size is closely related to the annealing temperature. Also, it was recently^45^ noticed that in perovskite systems, the average grain size increases on increasing the annealing temperature. Moreover, it was found that the bulk resistance is usually independent of the average grain size. However, the grain boundary resistance changes remarkably with the increase in the average grain size. Due to the congregation effect, both the grain size and crystallite size increase with the sintering temperature. The grain size increases due to the grain growth and the reduction in the grain boundary. Then, the grain boundary density is reduced. The minor grains become bonded together to connect more grains and consequently, this improves the compactness of the sample. Thus, the grains are powerfully linked together as the annealing temperature is increased. This development may be associated with the stronger diffusion process, where grain growth is endorsed due to the congregation effect at a higher annealing temperature. The latter leads to grain growth of the samples and provides better intergrain linking. Consequently, the grain boundary disorder effect is considerably reduced, causing a scattering effect on the grain boundary and hence affecting its resistivity. When the average grain size increases, the grain boundary density per volume unit is reduced. As a result, electrical resistivity decreases as the grain size is increased. The increase in the crystallite size with the increase in annealing temperature is the main cause behind the increase in DC conductivity.^47^

**Table 2:** Activation energy for Ni_0.6_Zn_0.4_Fe_1.5_Al_0.5_O_4_ at different annealing temperatures

	Ni_0.6_Zn_0.4_Fe_1.5_Al_0.5_O_4_ annealed at 600 °C	Ni_0.6_Zn_0.4_Fe_1.5_Al_0.5_O_4_ annealed at 900 °C	Ni_0.6_Zn_0.4_Fe_1.5_Al_0.5_O_4_ annealed at 1200 °C
*E*_a1_ (eV)	0.77		
*E*_a2_ (eV)	1.95	1.51	0.475
*E*_a3_ (eV)	0.232	0.159	0.151

The sample annealed at the highest temperature (1200 °C) exhibited the highest conductivity and the lowest activation energy and consequently the lowest defect density. This result is in good agreement with that reported in the literature.^48^ For each sample, along the studied range of temperature, we observed more than one value of activation energy. This suggests a change in the ease^49^ with which the conduction occurs as the temperature increases and this may be related to a change in the process assuring conduction. At a low temperature, the low values of activation energy are due to conduction by hopping.^39^ In contrast, the very high values (>1 eV) are associated with intrinsic conduction,^50^ indicating the transfer of electrons from the valence band to the conduction band. The intermediate values of activation energy (>0.4 eV) are linked to polaron hopping.^39^

### 3.3 AC conductivity

**Fig. 6:**
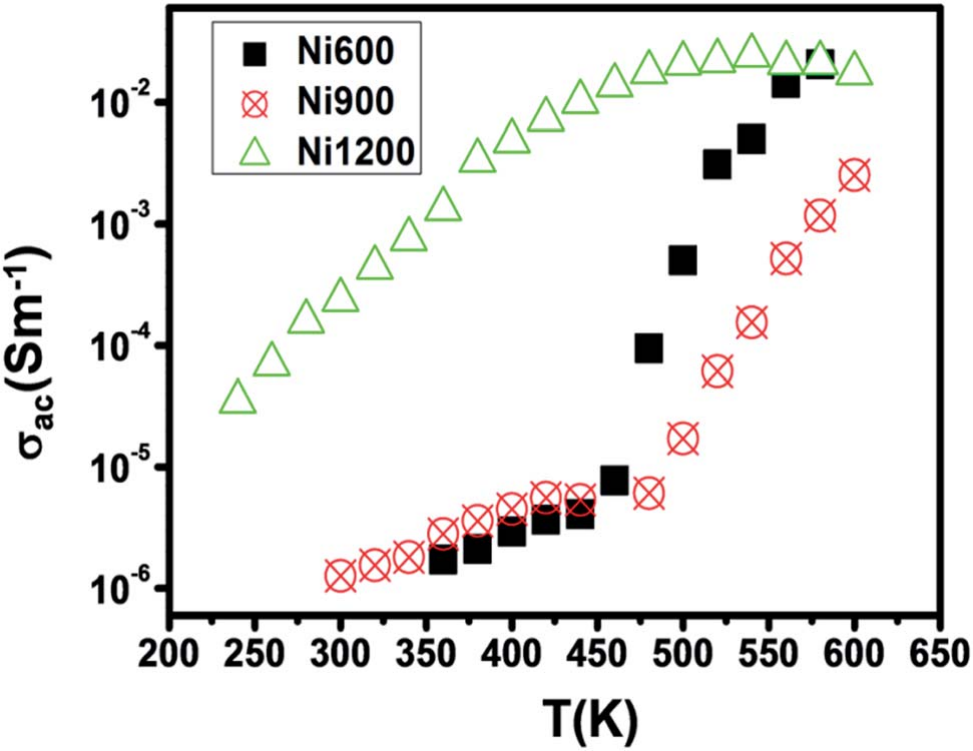
Variation of AC conductivity with temperature at 10^4^ Hz for Ni_0.6_Zn_0.4_Fe_1.5_Al_0.5_O_4_ annealed at 600 °C, 900 °C, and 1200 °C.

**Fig. 7:**
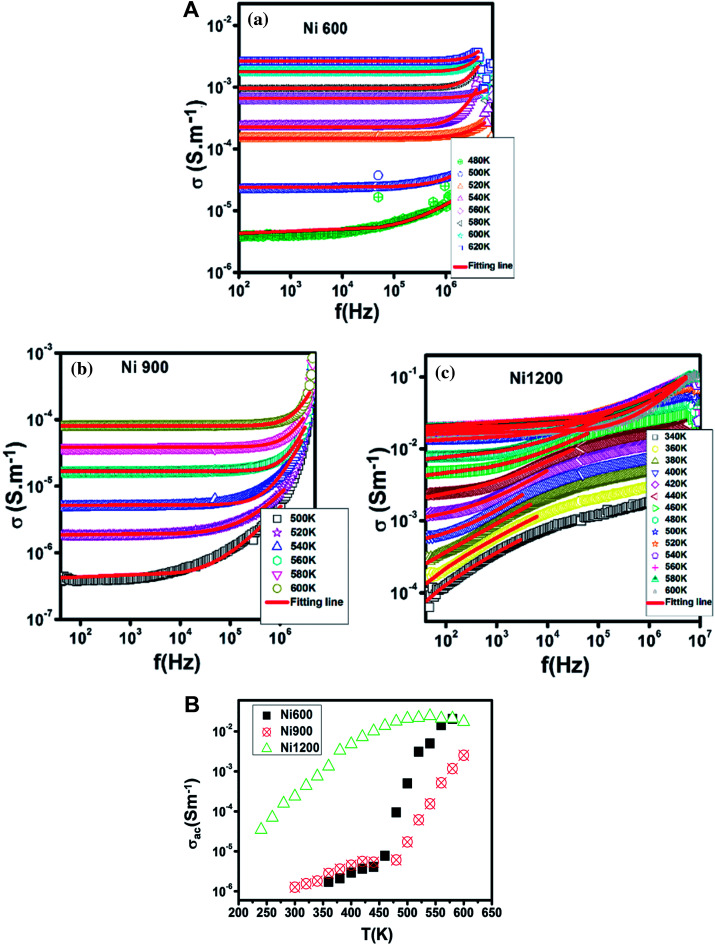
(A) Variation of the conductivity with frequency at different temperatures for Ni_0.6_Zn_0.4_Fe_1.5_Al_0.5_O_4_ annealed at 600 °C (a), 900 °C (b) and 1200 °C (c) with the fitting line. (B) Variation of the exponent ‘*s*’ with temperature for Ni_0.6_Zn_0.4_Fe_1.5_Al_0.5_O_4_ annealed at 600 °C, 900 °C, and 1200 °C.

The increase in Fe^2+^ concentration with the increase in annealing temperature^37^ explains the increase in conductivity with the increase in annealing temperature shown in Fig. 6 at low temperatures. This kind of variation was also found by Ravinder^37^ previously in Ni–Zn ferrites annealed at 1100 °C and 1250 °C. At a high temperature (from 460 K), the Ni900 sample has a minimal value of conductivity. To identify the conduction mechanism, the evolution of the exponent ‘*s*’ with temperature was plotted (Fig. 7(B)). The values of the exponent ‘*s*’ were deducted from the fit of the conductivity spectrum (Fig. 7(A)), whose evolution obeys the Jonscher power law:^51^*σ*_AC_ = *σ*_DC_ + *Aω*^*s*^

For the Ni600 and Ni900 samples, ‘*s*’ increased with temperature, which proved that the non-overlapping small polaron tunneling (NSPT) model ensures conduction. In contrast, for Ni1200, ‘*s*’ decreased, reached a minimum and then increased; thus, overlapping large polaron tunneling (OLPT) ensured conduction for this latter sample.^52^ For the Ni600 and Ni900 samples, the values of ‘*s*’ exceeded 1, while for the Ni1200 sample, the values of ‘*s*’ were lower than 1. Thus, for Ni600 and Ni900, jump of the charge carriers occurred between neighboring sites, whereas for Ni1200, the electron jump occurred between two distant sites.^53^

The hopping of charge carriers between ions with different valence states such as Fe^2+^, Fe^3+^ and Ni^2+^, Ni^3+^ in the octahedral sites is the mechanism responsible for AC conductivity:^35,36,38^ the hopping of electrons between Fe^2+^ and Fe^3+^ manifests n-type conduction^54,55^ and the transfer of holes between Ni^3+^ and Ni^2+^ represents p-type conduction.^56^

Two factors determine the probability of hopping: the activation energy and the distance between the ions implied in the conduction, which is called the jump length. The distance between two metals, *i.e.*, one localized at an A site and the other at a B site is larger than that when the two are localized in the B sites; thus, the hopping probability between A and B sites is lower as compared to that in the same B site.^36,54,57^

**Fig. 8:**
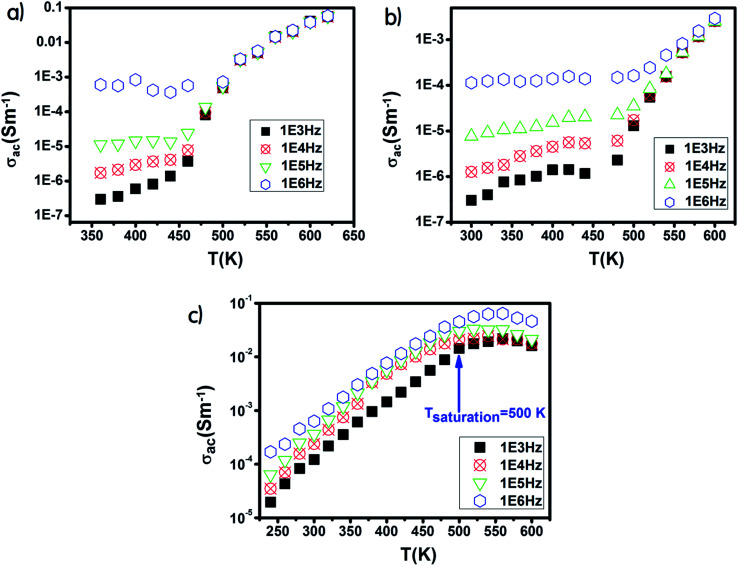
Temperature dependence of AC conductivity for Ni_0.6_Zn_0.4_Fe_1.5_Al_0.5_O_4_ annealed at 600 °C (a), 900 °C (b), and 1200 °C (c).

The Ni600 and Ni900 samples exhibited increase in conductivity on increasing frequency at a lower temperature, as shown in Fig. 8(a) and (b), while the conductivity of Ni1200 showed the same evolution along the entire explored range of temperature (Fig. 8(c)). This increase is a result of the pumping force. This force activates the emptying of trapping centers and the transfer of charge carriers between ions having different valence states.^58^ At high temperatures, the conductivities of the Ni600 and Ni900 samples were independent of frequency, which may be employed in adequate applications.

### 3.4 Impedance measurement

**Fig. 9:**
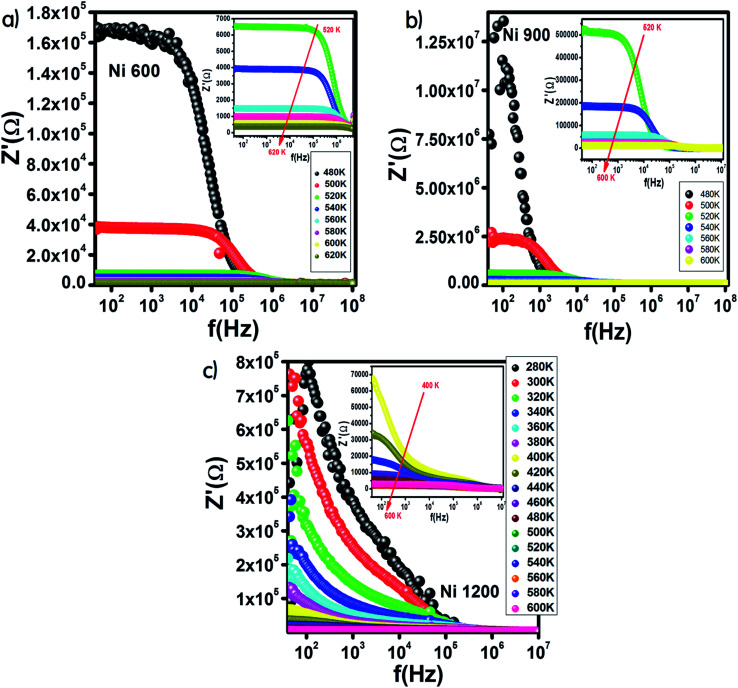
Variation of the real part of impedance with frequency at different temperatures for Ni_0.6_Zn_0.4_Fe_1.5_Al_0.5_O_4_ annealed at 600 °C (a), 900 °C (b), and 1200 °C (c).

Fig. 9 shows the variation in the real part of impedance *Z*′ with frequency. For the Ni600 and Ni900 samples (Fig. 9(a) and (b)), *Z*′ shows a similar behaviour with frequency and temperature. At low frequencies, *Z*′ has high values; by increasing the temperature and frequency, *Z*′ decreases, which indicates enhancement in conductivity. This evolution may be attributed to the improvement in drift mobility and the reduction in the trapped carrier density since both high temperature and high frequency induce the liberation of charge carriers from different trapping centers. At high frequencies, the values of *Z*′ were merged, which confirmed the existence of a space charge zone in our material.^59^ A comparable behavior has been observed in the literature.^60,61^ For the Ni1200 sample, *Z*′ decreased quickly with frequency (Fig. 9(c)) because the defect density was lower than that of Ni600 and Ni900; thus, the conductivity increased faster, which was confirmed by the decrease in resistivity.

**Fig. 10:**
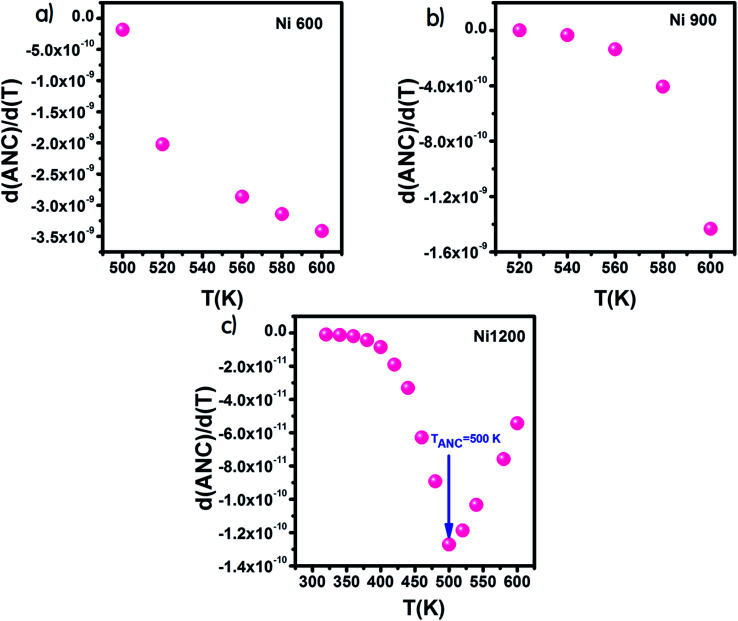
Variation of d(ANC)/d(*T*) with temperature for Ni_0.6_Zn_0.4_Fe_1.5_Al_0.5_O_4_ annealed at 600 °C (a), 900 °C (b), and 1200 °C (c).

From the curve of the variation in the real part of impedance with frequency, we can extract the variation in ANC, which is the abbreviation of average normalized change,^62^ and it indicates the intrinsic response of a material. Then, we presented the variation of d(ANC)/d(*T*) as a function of temperature (Fig. 10). The sample annealed at 1200 °C exhibited a special temperature, at which modification of the curve slope occurred. This temperature corresponded to the temperature at which all the trapping centers become empty. For Ni1200, 500 K was enough to liberate all the trapped charge carriers, which contributed to conduction. This temperature is the same as the temperature of saturation extracted from the plot *σ* = *f*(*T*). In contrast, for Ni600 and Ni900, even 600 K was not sufficient to empty all the centers, and some charge carriers were still frozen and needed to be liberated at higher temperatures.

**Fig. 11:**
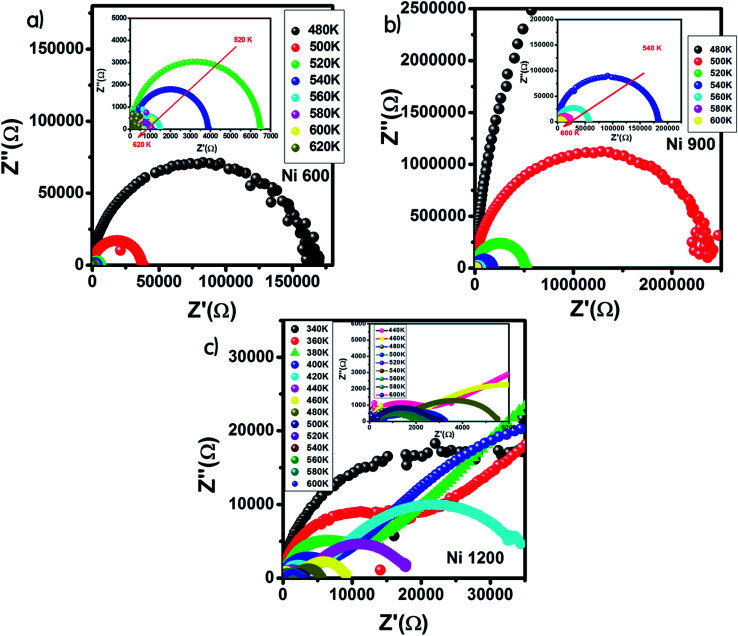
Nyquist diagram for Ni_0.6_Zn_0.4_Fe_1.5_Al_0.5_O_4_ annealed at 600 °C (a), 900 °C (b), and 1200 °C (c).

**Fig. 12:**
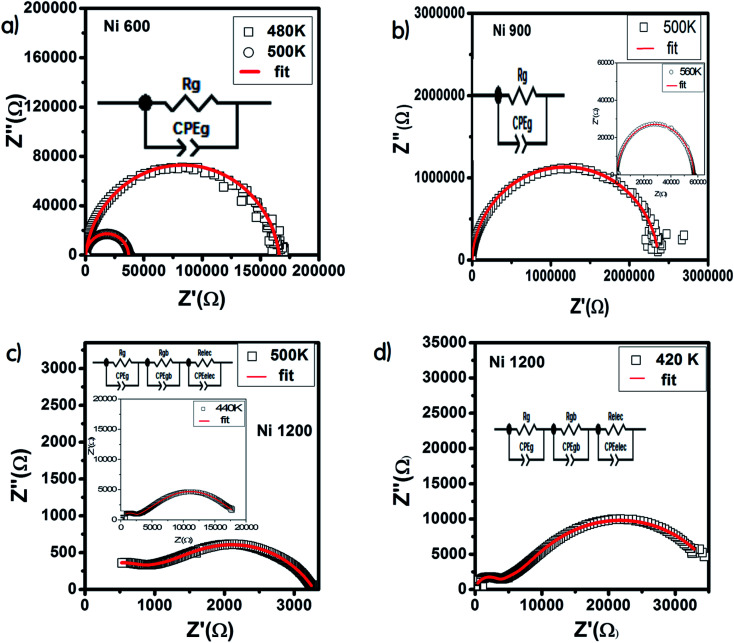
Results of fitting of the Nyquist diagram and equivalent circuit model for Ni_0.6_Zn_0.4_Fe_1.5_Al_0.5_O_4_ annealed at 600 °C (a), 900 °C (b), and 1200 °C (c and d).

The Nyquist diagram offers an opportunity to examine the contribution of the grains and grain boundary to conduction and the modeling of each sample by an equivalent electrical circuit. For the Ni600 and Ni 900 samples, the Nyquist diagrams (Fig. 11(a) and (b)) were formed by a single semi-circle and could be modeled by the resistance of the grains branched in parallel to the capacity of the grains (Fig. 12(a) and (b)), as reported in the literature; the single semi-circle indicates the existence of the grain effect.^35^ The decrease in the diameter of the semi-circle with temperature was caused by the reduction in grain resistance;^63^ this kind of evolution of resistance was confirmed by the fitting parameters and it is in accordance with the behavior of conductivity seen previously.

**Fig. 13:**
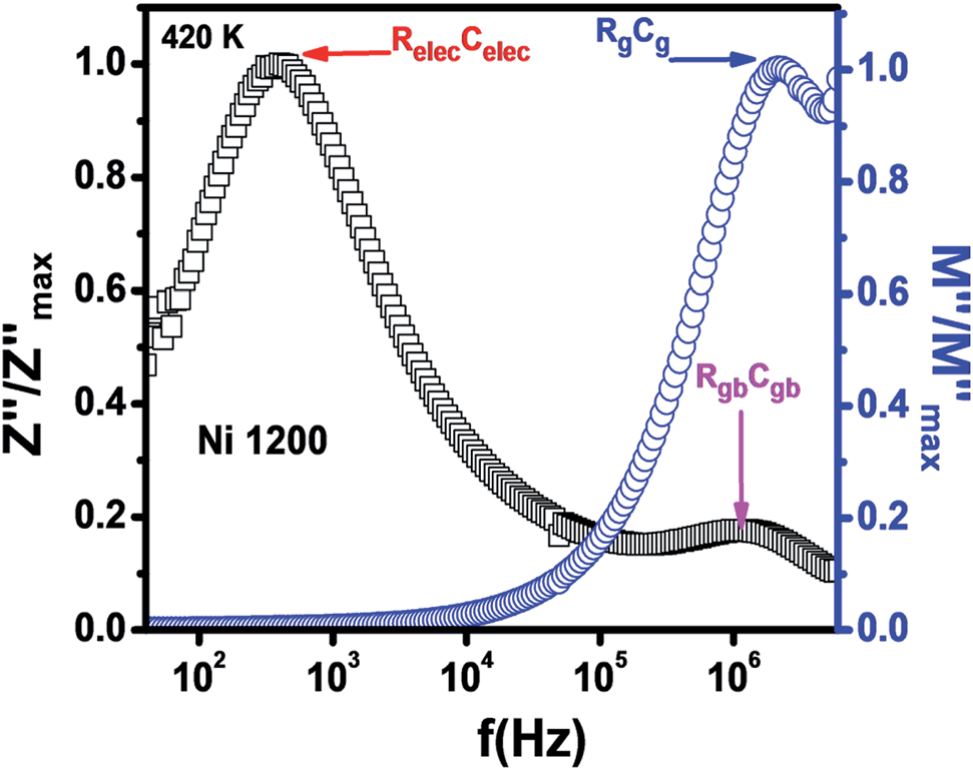
Combined normalized *Z*′′ and normalized *M*′′ *versus* frequency plots at 420 K for Ni_0.6_Zn_0.4_Fe_1.5_Al_0.5_O_4_ annealed at 1200 °C.

The Nyquist diagram (Fig. 11(c)) of the Ni1200 sample is composed of three semi-circles: an incomplete semi-circle at higher frequencies presents the grain contribution; the second semi-circle at the middle presents the grain boundary contribution,^35^ and the third semi-circle at lower frequencies is ascribed to the electrode effect (Fig. 12(c) and (d)). The two latter semi-circles overlapped and deconvolution was thus necessary. In order to ensure the occurrence of these three effects, we depict the variation of the imaginary part of the electric modulus and the imaginary part of impedance with frequency at 420 K (Fig. 13) for this sample. As found in the literature,^64^ the peak that appears in the modulus plot at a high frequency is ascribed to the bulk (grain) effect, while the two other peaks in the impedance plot are attributed to the grain boundary and electrode effect.

**Fig. 14:**
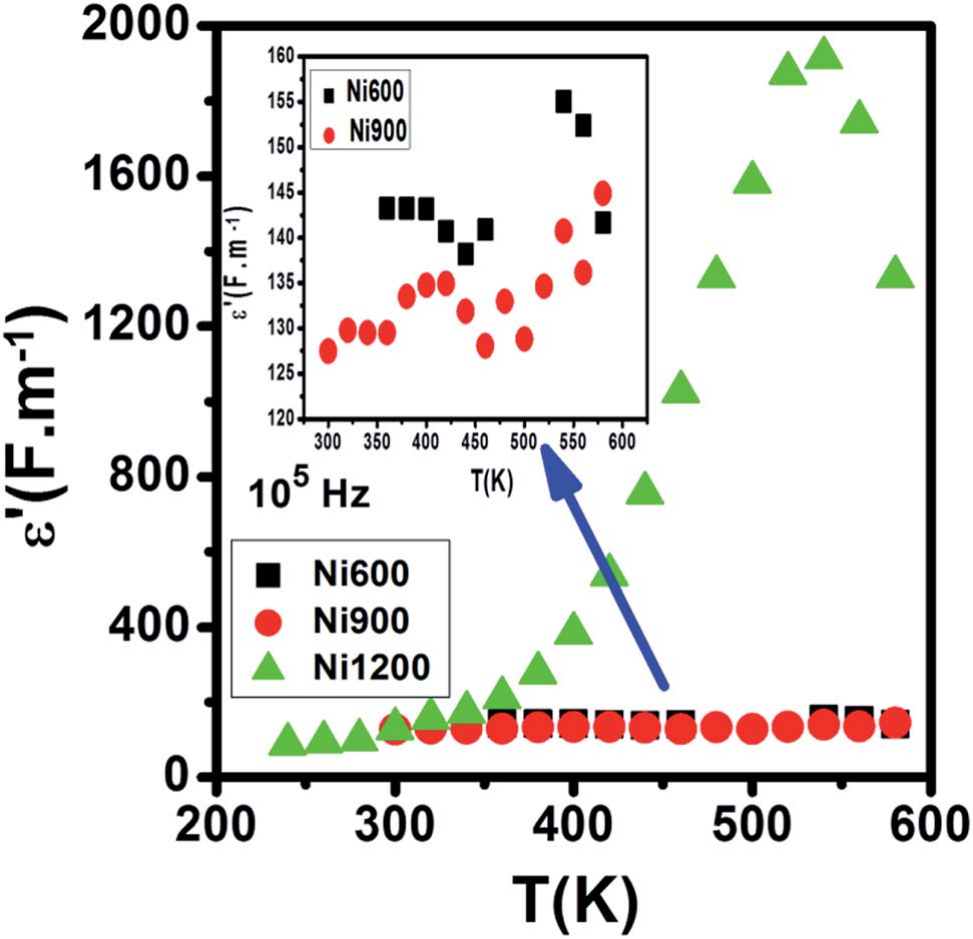
Evolution of the dielectric constant with temperature at 10^5^ Hz for Ni_0.6_Zn_0.4_Fe_1.5_Al_0.5_O_4_ annealed at 600 °C, 900 °C, and 1200 °C.

Compared to the grain boundary effect in Ni600 and Ni900, the emergence of the grain boundary effect in Ni1200 can be explained by the fact that with the increase in annealing temperature, the contribution of the grain boundary to the total resistance increases, as reported in the literature.^39^ This may be due to the structural modification manifested by the increase in grain size because of the higher annealing temperature; thus, normally, the interior grain conduction dominates.^39^ However, in our case, as shown in Table 3, with the annealing temperature increasing from 600 °C to 900 °C, the grain resistance increases. This may be due to the resistive pores intercalated inside the newly formed grain during the fusion of adjacent grains because of the annealing process.^65–67^ In order to demonstrate that Ni900 contained pores, the variation in the dielectric permittivity with temperature was plotted (Fig. 14). Verma *et al.* showed that the sample with the most porous structure has the lowest dielectric constant,^27^ which was Ni900 in the present study. This seems at first sight not congruent with the structural results, which suggest that Ni600 is the most porous sample; however, these later analyses were conducted at room temperature, while the dielectric plot extended all over the explored range of temperatures of the measurements, which may affect the porosity and cause its modification above room temperature. Then, at the annealing temperature of 1200 °C, the grain resistance decreased due to the evacuation of these pores.^65,66,68^ Besides, for this later sample, the resistance of the grain boundary was higher as compared to the resistance of the grains; this observation is also presented in another paper,^36^ where the grain boundary is more resistive than the grain.

**Table 3:** Fitting parameters of the Nyquist diagram at 500 K for Ni_0.6_Zn_0.4_Fe_1.5_Al_0.5_O_4_ at different annealing temperatures

500 K			
	Ni_0.6_Zn_0.4_Fe_1.5_Al_0.5_O_4_ annealed at 600 °C	Ni_0.6_Zn_0.4_Fe_1.5_Al_0.5_O_4_ annealed at 900 °C	Ni_0.6_Zn_0.4_Fe_1.5_Al_0.5_O_4_ annealed at 1200 °C
Grain resistance (Ω)	3.7 × 10^4^	2.37 × 10^6^	8.42 × 10^2^
Grain fractal capacity (F)	6.3 × 10^−11^	5.6 × 10^−11^	1.2 × 10^−9^
Exponent ‘*α*’(grain)	0.956	0.971	0.788
Grain boundary resistance (Ω)			2.3 × 10^3^
Grain boundary fractal capacity (F)			4.2 × 10^−7^
Exponent ‘*α*’(grain boundary)			0.612
Electrode resistance (Ω)			1.69 × 10^2^
Electrode fractal capacity (F)			1.15 × 10^−8^
Exponent ‘*α*’ (electrode)			0.89

### 3.5 Dielectric behavior

**Fig. 15:**
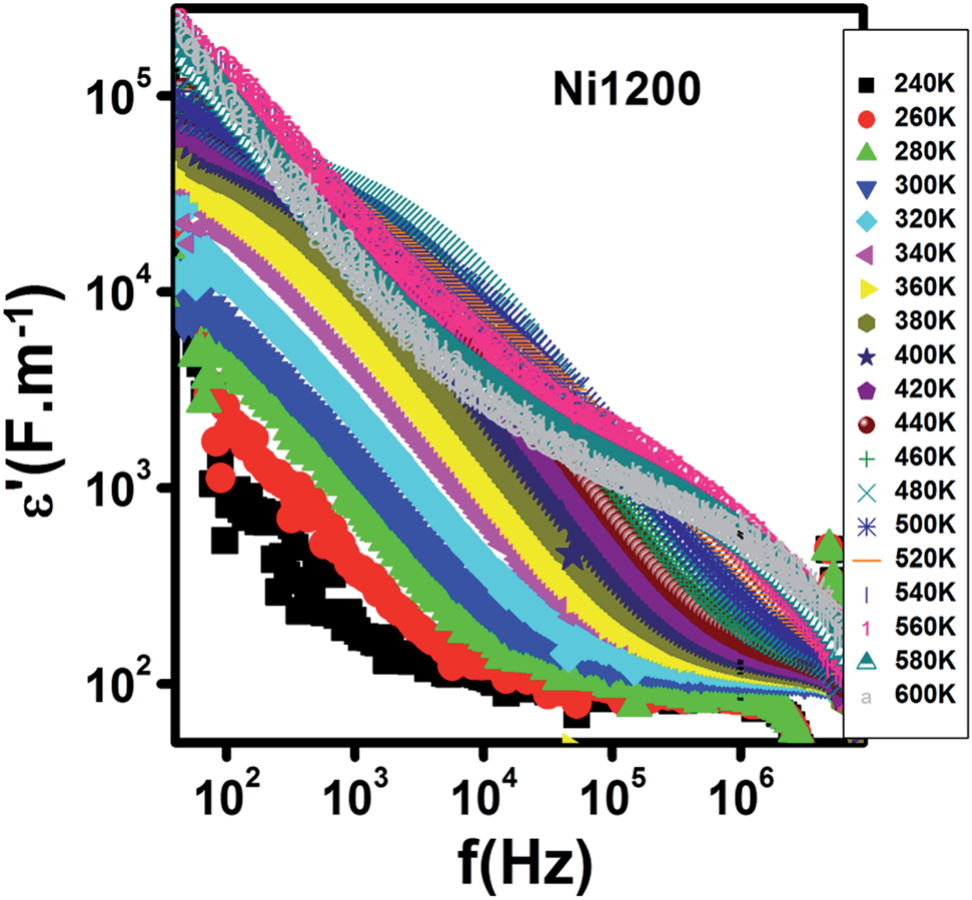
Spectrum of the real part of the dielectric permittivity at different temperatures for Ni_0.6_Zn_0.4_Fe_1.5_Al_0.5_O_4_ annealed at 1200 °C.

Fig. 15 represents the variation of the real part *ε*′of the dielectric constant with frequency for the Ni1200 sample. It was detectable that *ε*′ has large values at low frequencies. Then, it decreases. Its magnitude and behavior are similar to that of Ni_0.5_Zn_0.5_Cr_0.5_Fe_1.5_O_4_ nanoparticles dielectrically studied by Tan *et al.*^69^ This evolution was due to the decrease in polarization with frequency.^70^ Four kinds of polarizations, namely, interfacial, dipolar, electronic, and ionic polarization have an obvious effect on the dielectric constant.^71^ These polarizations' contribution declines with frequency.^71^ Besides, it is clear that at low frequencies, *ε*′ is temperature-dependent; however, at high frequencies, it merges to the same value and becomes independent of temperature. This behavior may be explained as follows: the contribution of dipolar and interfacial polarizations dominates at low frequencies and these two types of polarizations depend strongly on temperature. In contrast, at high frequencies, the electronic and ionic polarizations contribute effectively.^71^

**Fig. 16:**
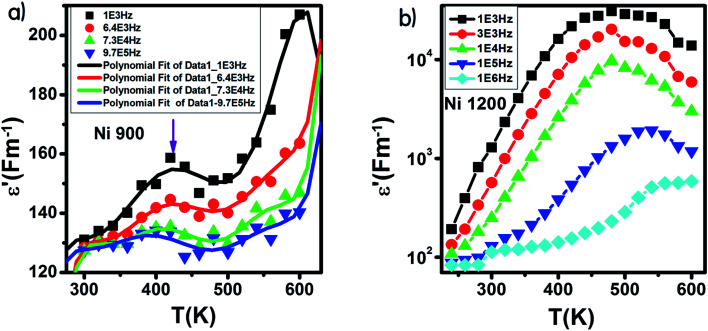
Variation of the real part of the dielectric permittivity with temperature in an AC regime for Ni_0.6_Zn_0.4_Fe_1.5_Al_0.5_O_4_ annealed at 900 °C (a) with fitting line and at 1200 °C (b).

**Fig. 17:**
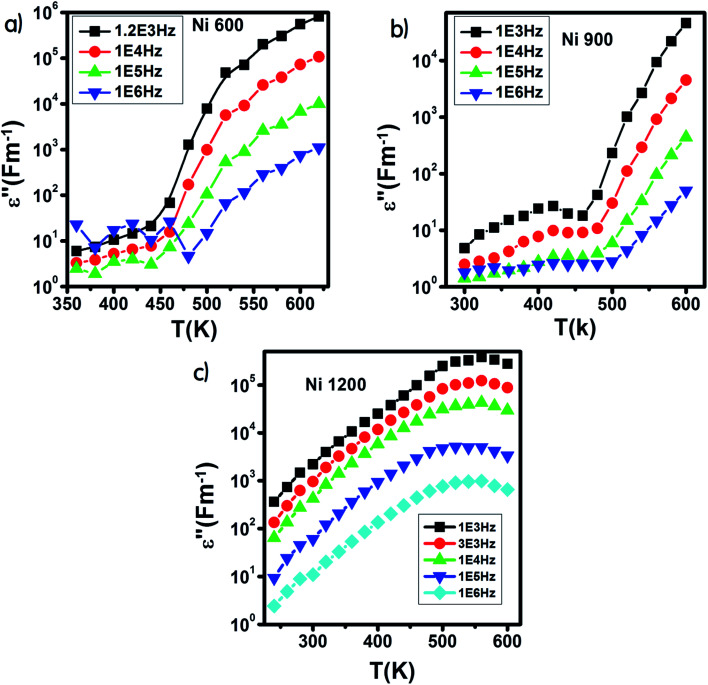
Variation of the imaginary part of the dielectric permittivity with temperature in an AC regime for Ni_0.6_Zn_0.4_Fe_1.5_Al_0.5_O_4_ annealed at 600 °C (a), 900 °C (b), and 1200 °C (c).

The variation of *ε*′ with temperature is shown in Fig. 16. The increase at the beginning of *ε*′ with temperature may be understandable by the increase in polarization caused by the increase in temperature, which brings thermal energy to the samples.^71^ The Ni1200 sample (Fig. 16(b)) can be distinguished by the appearance of a peak at a particular temperature because at this special temperature, the jumping frequency of an electron between Fe^2+^ and Fe^3+^ becomes equivalent to the applied field frequency.^70,72^ To clarify this idea, we can say that at first, the frequency of hopping tries to reach the frequency of the applied field; thus, the polarization increases gradually. It reaches a maximum when the two frequencies are equal; then, the frequency of the applied field continues to increase, while the frequency of hopping cannot follow this increase; this results in decrease in polarization and thus decrease in the dielectric constant. This phenomenon is also shown by the variation in the imaginary part of dielectric permittivity *ε*′ with temperature in the AC regime (Fig. 17). Some other research attributed this peak to the matching between the thermal energy and the energy due to the frequency of the natural oscillation of electric dipoles.^71^

For the Ni900 sample, it is important to notice the appearance of an anomaly peak at 426 K in the variation of the real part of dielectric permittivity *versus* temperature (Fig. 16(a)). A similar anomaly was found by Mohamed *et al.*^73^ for a nanocomposite PANI-Ni_0.5_Zn_0.5_Fe_1.5_Cr_0.5_O_4_ around 333 K and by Tan *et al.*^74^ at 305 K. This peak may be attributed to local polarization^75^ and its wide dispersion indicates the diffuse character of phase transition that the material undergoes.^76^

With increasing frequency, the dielectric maxima shift to higher temperatures. This behavior is a direct result of the fact that the restoration of polarization needs higher temperatures because the time lag is higher. This is caused by the inability of charge carriers to follow the pace of the fast-changing field at high frequencies.^77,78^

The examination of the order of magnitude of the dielectric constant may confirm the suitability of the studied samples for use in electro-optic and photonic applications due to the low values at high frequencies^79^ for the sample annealed at 1200 °C and at all the explored frequencies for the sample annealed at 900 °C.

## 4. Conclusion

The physical properties of Ni_0.6_Zn_0.4_Fe_1.5_Al_0.5_O_4_, annealed at 600 °C, 900 °C, and 1200 °C were investigated. The presence of a single-phase cubic spinel structure with the *Fd*3̄*m* space group was confirmed by X-ray diffraction patterns. The SEM images of the investigated nanoparticles demonstrated that the samples were nano-sized. The increase in annealing temperature led to an increase in crystallite size and the agglomeration rate. The electrical characterization of Ni_0.6_Zn_0.4_Fe_1.5_Al_0.5_O_4_ annealed at 600 °C, 900 °C, and 1200 °C allowed us to conclude that the increase in temperature improved the electrical conductivity along the entire explored range for Ni600 and Ni900, while this improvement declined after 500 K for Ni1200, where a metallic behavior appeared. The sample annealed at 1200 °C possessed the highest conductivity and the lowest activation energy. The conduction mechanism was the hopping of charge carriers between ions of different valence states. The impedance plots were in good agreement with the conductivity plots and confirmed the emergence of grain boundary and electrode effects with the increase in annealing temperature. The dielectric behavior was correlated to the polarization effect. A specific behavior of the sample annealed at 900 °C appeared with an anomaly peak, indicating the diffuse character of a phase transition that the material undergoes. This latter sample is a good candidate for electro-optic and photonic applications.

## Conflicts of interest

There are no conflicts to declare.

## Supplementary Material
